# Electrophysiology‐aided DBS targeting the ventral intermediate nucleus in an essential tremor patient with MRI‐incompatible lead: A case report

**DOI:** 10.14814/phy2.15730

**Published:** 2023-10-03

**Authors:** Stefanie Glowinsky, Hagai Bergman, Omer Zarchi, Shlomo Fireman, Johnathan Reiner, Idit Tamir

**Affiliations:** ^1^ The Edmond and Lily Safra Center for Brain Sciences Hebrew University Jerusalem Israel; ^2^ Department of Medical Neurobiology Hebrew University Jerusalem Israel; ^3^ Department of Neurosurgery Hadassah Medical Center, Hebrew University Jerusalem Israel; ^4^ Intraoperative Neurophysiology Unit Rabin Medical Center, Beilinson Hospital Petach Tikvah Israel; ^5^ Department of Anesthesiology Rabin Medical Center, Beilinson Hospital Petach Tikvah Israel; ^6^ Department of Neurology Rabin Medical Center, Beilinson Hospital Petach Tikvah Israel; ^7^ Department of Neurosurgery Rabin Medical Center, Beilinson Hospital Petach Tikvah Israel

**Keywords:** deep brain stimulation, electrophysiology, essential tremor, thalamus, ventral intermediate nucleus

## Abstract

Essential tremor (ET) is a common disease in the elderly population. Severe, medication‐refractory ET may require surgical intervention via ablation or deep brain stimulation (DBS). Thalamic Vim (Ventral intermediate nucleus), targeted indirectly using atlas‐based coordinates, is the classical target in these procedures. We present a case of an ET patient with a non‐MR‐compatible cardiac orphaned leads who was a candidate for DBS surgery. Due to the lead constraints of MR use, we used a head computed tomography (CT) with contrast media as the reference exam to define the AC, PC, and midline, and to register and indirectly target the Vim. For target validation, we used intraoperative electrophysiological recordings and intraoperative CT. We implanted bilateral directional leads at the target location. We used the‐essential‐tremor‐rating‐assessment‐scale (TETRAS) pre and postoperatively to clinically evaluate tremor. Intraoperative micro‐electrode recordings (MERs) showed individual tremor cells and a robust increase in normalized root mean square (NRMS) indicating entry to the Vim. Postoperative visualization using lead‐DBS along with dramatic clinical improvements show that we were able to accurately target the Vim. Our results show that CT‐only registration and planning for thalamic Vim DBS is feasible, and that MERs and intraoperative CT are useful adjuncts for Vim target validation.

## 1 BACKGROUND

Essential tremor (ET) is a common disease in the elderly population (Louis et al., 1998) and is typically treated pharmacologically. However, many patients with severe, refractory ET require surgical intervention either via ablation or DBS (Elble et al., 2018). The Vim nucleus of the thalamus first emerged as a potential target for ET‐DBS surgery due to its efficacy in reducing tremor upon stimulation during thalamotomies performed in the 1960s (Benabid et al., 1987; Hassler et al., 1960). Back in the previous century, thalamotomies were conducted using ventriculography to mark the AC and PC as the anterior and posterior borders of the third ventricle. Since the introduction of the MRI to routine clinical use, AC, PC, and midline are recognized on the MRI. As the Vim cannot be visualized on MRI, it is still being targeted indirectly today using AC‐PC coordinates, sometimes with assistance from intraoperative MERs to detect the thalamic borders and to differentiate the motor thalamus (Vim) from neighboring areas (Garonzik et al., 2002). In the reported case, the patient remains with MRI‐incompatible orphaned leads from a cardiac pacemaker that was implanted 20 years ago. Therefore, we chose to target the Vim using CT only registration and indirect targeting aided by MERs. Rather than using classical physiological navigation, a time‐consuming procedure which uses the spiking activity of well‐isolated single units to ensure location (Gross et al., 2006), we chose to use a more recent, alternative method. This method uses the multi‐unit activity with high spatial sampling (0.1 mm intervals) along the trajectory and short time duration recording (4–8 s) in each site (Moran et al., 2008). The former has been used successfully in detecting the border and subdomains of the STN (Moran et al., 2008) and of the globus pallidus internus (GPi) (Valsky et al., 2020); here we report using this technique for thalamic Vim navigation in an ET patient.

## 2 HISTORY

Our patient is a right hand dominant 75‐year‐old male. Eight years prior to surgery he developed bilateral hand tremor, mild leg tremor, and imbalance, which worsened gradually. He was initially diagnosed with Parkinson's disease and treated with levodopa, but the symptoms were non‐responsive to medications (UPDRS decrease from 35 to 29, resistant action tremor), and a F‐DOPA scan was negative. Therefore, the diagnosis changed to ET plus. However, a trial of beta blockers (propranolol, 80 mg) failed to significantly improve his tremor, and he did not tolerate the anticonvulsant primidone due to induced sleepiness. On preoperative examination, he had bilateral symmetric positional and intentional tremor with mild rest tremor, mild cogwheel rigidity, and bradykinesia. His TETRAS score was 66.5, showing significant effect on ADL. He was therefore approved for DBS surgery.

## 3 METHODS

### 3.1 Planning

We obtained written informed consent from the patient for the study. We used high‐resolution contrast CT (navigation protocol; 1 mm slices, no gaps, no overlap) for registration and indirectly targeted the Vim using AC‐PC coordinates (AC‐PC distance: 30 mm) and preoperative CT scan using the following stereotactic coordinates in relation to mid‐commissural point, MCP (henceforth referred to as the pre‐planned target location). On the left side: *X* = −14.5, *Y* = −7.5, and *Z* = 0, 61.6 degrees from the axial and 20.5 degrees from the mid‐sagittal planes. On the right side: *X* = 14, *Y* = ‐7.2, and *Z* = 0, 61.6 degrees from the axial and 21 degrees from the mid‐sagittal planes. The Vim lead target was 11 mm from the lateral wall of the third ventricle bilaterally. In the morning of surgery, after fixation of the CRW stereotactic frame and fiducial box on the patient's head (under local anesthesia and mild sedation) the patient underwent a second CT for registration. Stealth 8 navigation system was used for image fusion, trajectory planning, and lead localization confirmation.

### 3.2 Anesthesia protocol

The patient underwent moderate sedation with a 20 mg bolus of propofol at the beginning of surgery for bilateral skin incision and burr hole drilling. The patient was kept awake during MER and test stimulation. Nicardipine (a calcium channel blocker) was given as a drip when necessary to keep the target systolic blood pressure less than 150 mmHg during the surgery for a total dosage of 6 mg. After implanting both leads, propofol was restarted at 1.4 mg/kg/h. Implantable pulse generator (IPG) insertion was conducted under general anesthesia.

### 3.3 Micro‐electrode recording

We recorded neural activity (single and multiunit) using Sonus microelectrodes connected to the Neuro‐omega system (Alpha‐Omega Engineering, Nof HaGalil, Israel), starting on the left. Data were sampled at 44 kHz and hardware filtered at 0.1–9000 Hz. We began recording at 15 mm above the target location, advancing the electrode in 100 μm steps, recording for 8 s per site.

### 3.4 Macro‐electrode stimulation

On both sides we stimulated the macro‐electrode at 3 mm above pre‐planned target, with increasing intensity (0.25 mA–4 mA), a pulse width of 60 μs, and a frequency of 130 Hz.

### 3.5 Implantation

We implanted the pulse generator and Vercise PC directional leads (Boston Scientific) with the lower edge of the deepest contact at the pre‐planned target on both sides. Intraoperative CT (O‐arm, Medtronic Inc.) was used to verify the final location of the leads.

### 3.6 Anatomy

To visualize the implanted leads postoperatively, we used Lead‐DBS (v2.5) (Horn & Kühn, 2015) to co‐register the patient's postoperative CT with the DISTAL atlas (Chakravarty et al., 2006; Ewert et al., 2018).

### 3.7 Electrophysiology

The electrophysiological data were processed intraoperatively. We applied a software filter from 500 to 2000 Hz and then calculated the normalized RMS (NRMS) and power spectral density plots (PSD) as described in (Zaidel et al., 2010). In the offline analysis we included the first 2 s of recording in each site since no major movement artifacts and cell injuries were noted in this surgery. We set the minimum value of the log‐transformed PSD values to −35.

### 3.8 Tremor cell analysis

Tremor cells (TCs) were identified online visually and by audio. We extracted an 80s extracellular TC recording with simultaneous EMG activity. We filtered the extracellular and EMG data between 400–2000 Hz and 50–500 Hz, respectively (fourth order, zero‐phase). Signals were rectified (Dakin et al., 2014), zero centered (Moran et al., 2008), and low‐pass filtered at 70 Hz (fourth order, zero phase). The extracellular signal was down‐sampled to 11 kHz to match the EMG sampling frequency. We calculated the auto‐correlation of each signal and the cross‐correlation of each pair of signals with a lag of up to ±500 ms and normalized these results by their maximum value. We calculated the power spectrum of each signal using the pwelch method (Matlab 2021b) with a 1 s Hanning window and an overlap of 50%. We normalized the power by the total power in the frequencies of interest (1–30 Hz) of the respective signal. We calculated the magnitude‐squared coherence between each pair of signals using the same windowing, overlap, and normalization technique.

### 3.9 EMG tremor activity analysis

We identified 66 recordings (minimum duration: 2 s, average duration: 12.163 s, 50 on the left side, 16 on the right side) with tremor activity in both the wrist flexor and extensor EMG. We filtered, rectified, and re‐filtered the EMG signals in the same manner as we did above (Section 3.8). We performed a cross‐correlation between each pair of signals with a lag ranging from −0.5 to 0.5 s. For each cross‐correlation result, we performed mean normalization and calculated the 95% confidence intervals.

## 4 RESULTS

**FIGURE 1:**
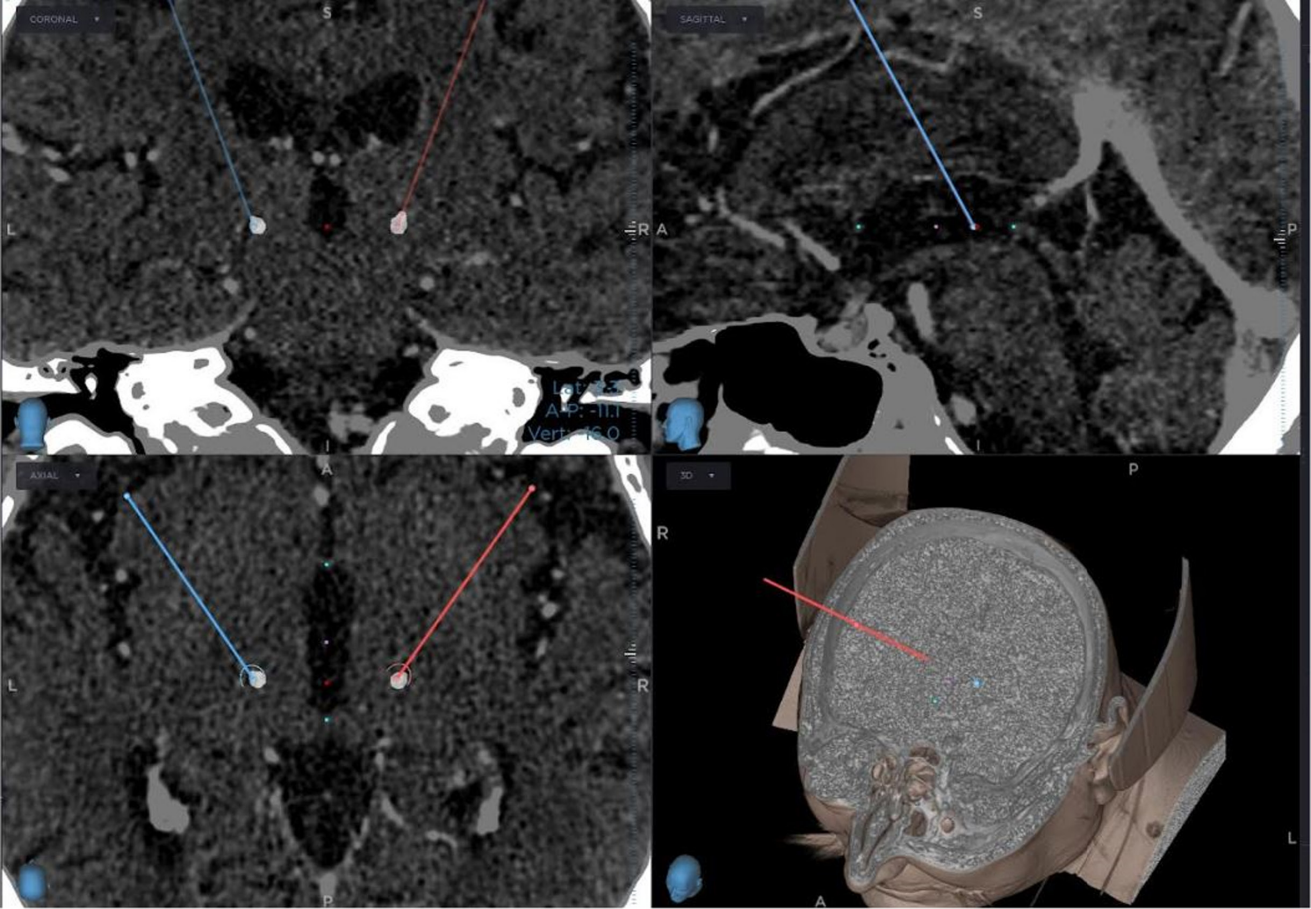
Co‐registration of preoperative CT with contrast media and postoperative CT. Electrodes targeting the Vim are shown in blue (left) and red (right) in the coronal (top left), sagittal (top right), axial (bottom left), and 3d (bottom right) views. The silver circles in the coronal and axial views mark the actual placement of the electrodes. In the sagittal and axial views, the anterior and posterior commissures can be seen (light blue) as well as the MCP (pink).

**FIGURE 2:**
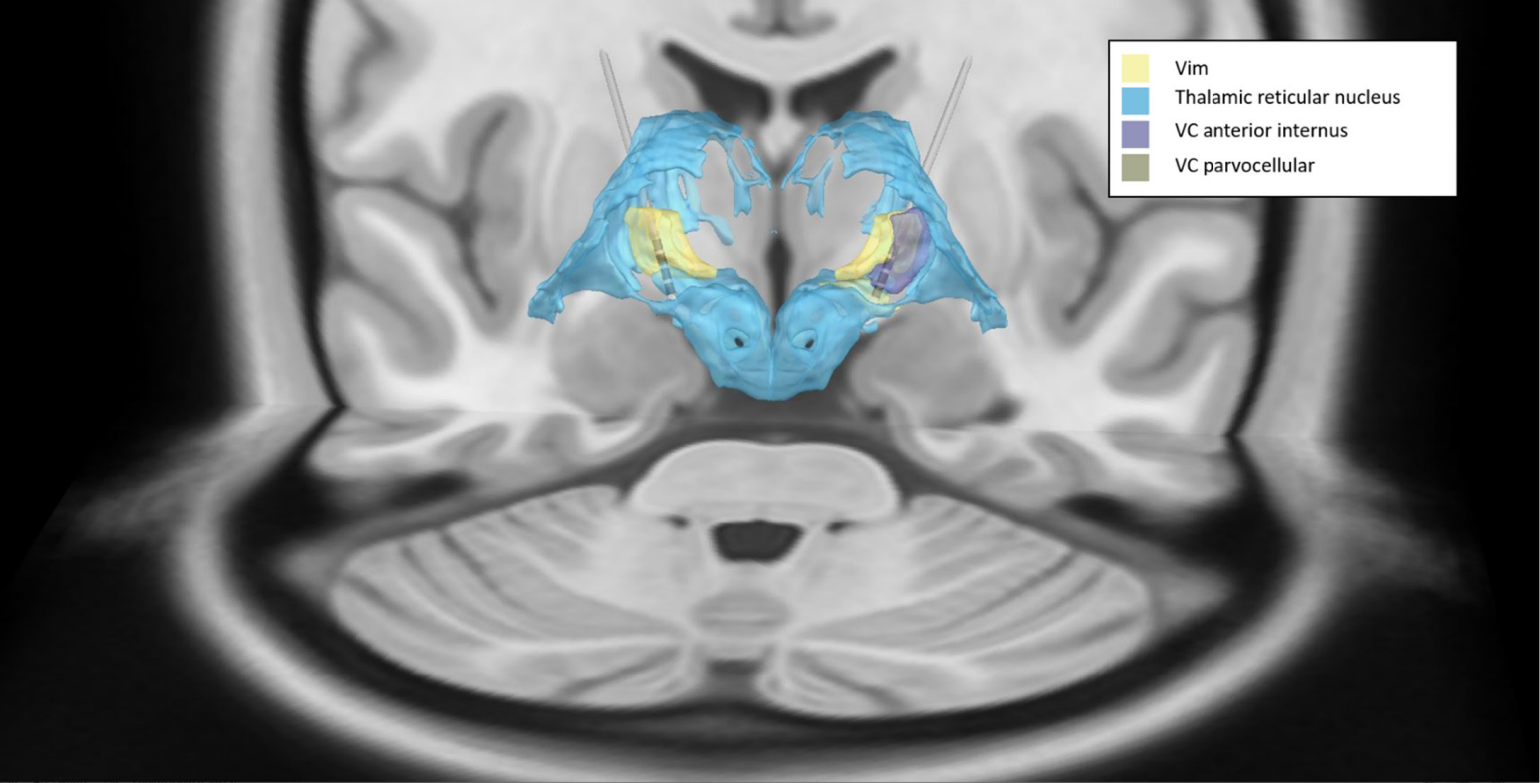
Implanted electrode location visualized using Lead‐DBS (posterior view). LEAD‐DBS results showing the location of the implanted leads.

**FIGURE 3:**
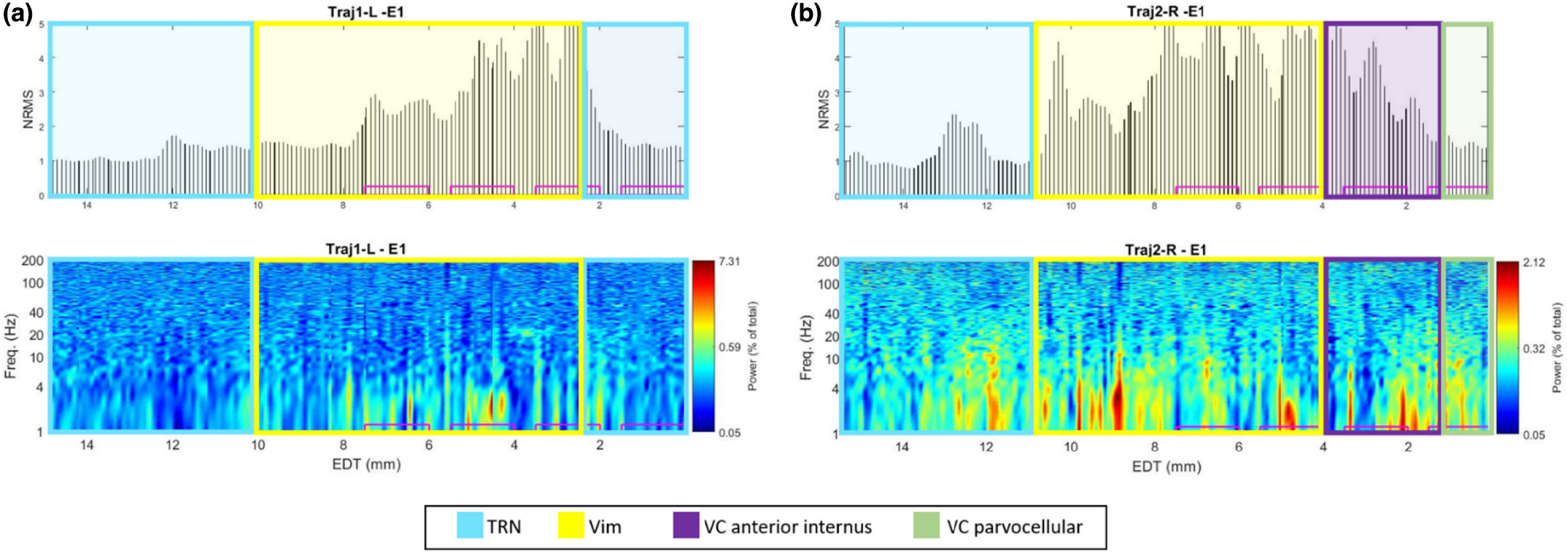
Physiological recording of the Vim trajectories. NRMS and PSD plots for (a) left (first) trajectory and (b) right (second) trajectory, starting at a depth of 15 mm above target. The colors show the locations of the thalamic regions we passed through based on Lead‐DBS results. The pink rectangles represent the contacts of the implanted leads.

The co‐registered pre and postoperative CT scans are shown in Figure 1. Postoperative analysis of the postoperative CT using Lead‐DBS co‐registered with the DISTAL atlas (Chakravarty et al., 2006; Ewert et al., 2018) shows the upper two contacts are located in the Vim on both sides (Figure 2). On the left, the lower two contacts are located in the thalamic reticular nucleus (TRN), whereas in the right they are located in the ventral caudal nucleus (Vc) (Figure 2). Both trajectories started inside the TRN (15 mm above target) and passed into the Vim at 10/11 mm (left/right), after which they passed back into the TRN at 2.5 mm (left) or the Vc at 4 mm (right) (Figure 3).

**FIGURE 4:**
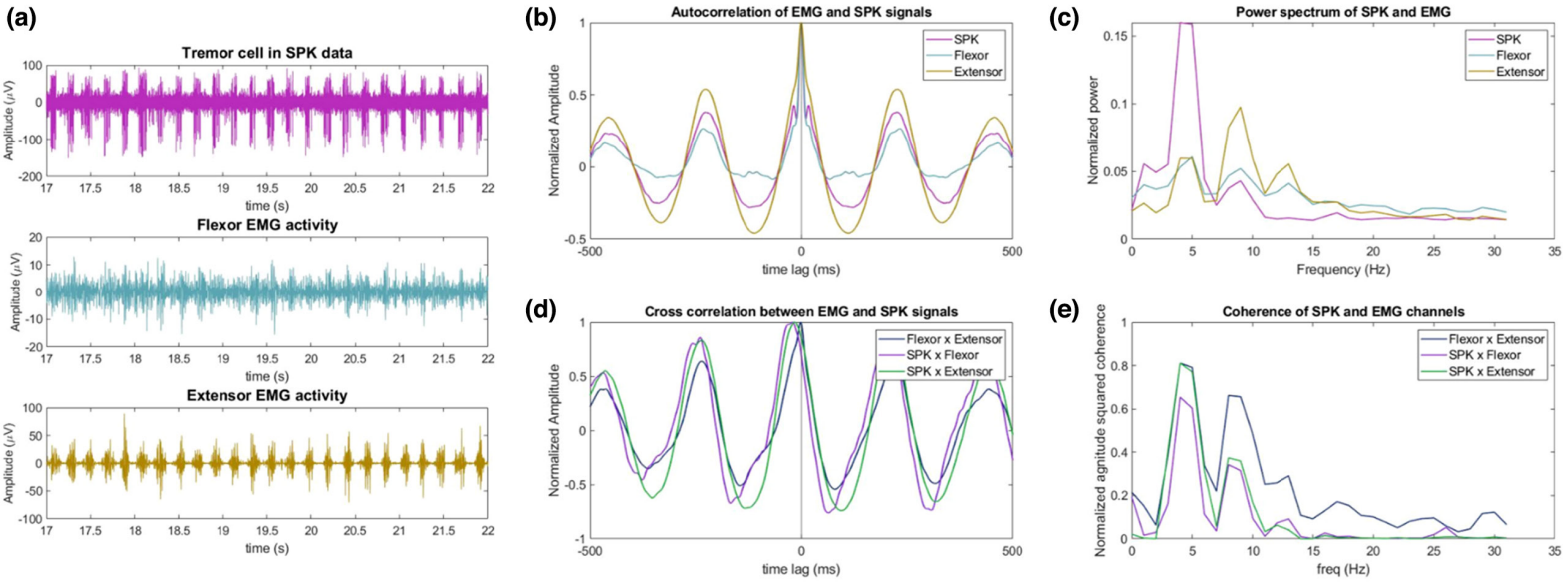
Electrophysiological and EMG activity of tremor cell. A Vim TC was recorded in the left trajectory with simultaneous EMG right wrist flexor and extensor activity. (a) Displays a 5 s segment of the TC/EMG signal, (b) autocorrelation of each signal, (c) power spectrum of each signal, (d) normalized cross correlation between each pair of signals, (e) the ms‐coherence of each pair of signals.

The electrophysiology of the MERs showed delta and theta activity (1–4 and 4–7 Hz, respectively) throughout the motor nuclei of the thalamus, as well as a steep and sustained rise in NRMS (except for the Vc parvocellular) (Figure 3). Intraoperatively, three tremor cells TCs were observed in the electrophysiology of the left trajectory (Figure 4) indicating that we were inside the Vim, but they were not observed in the right trajectory.

**FIGURE 5:**
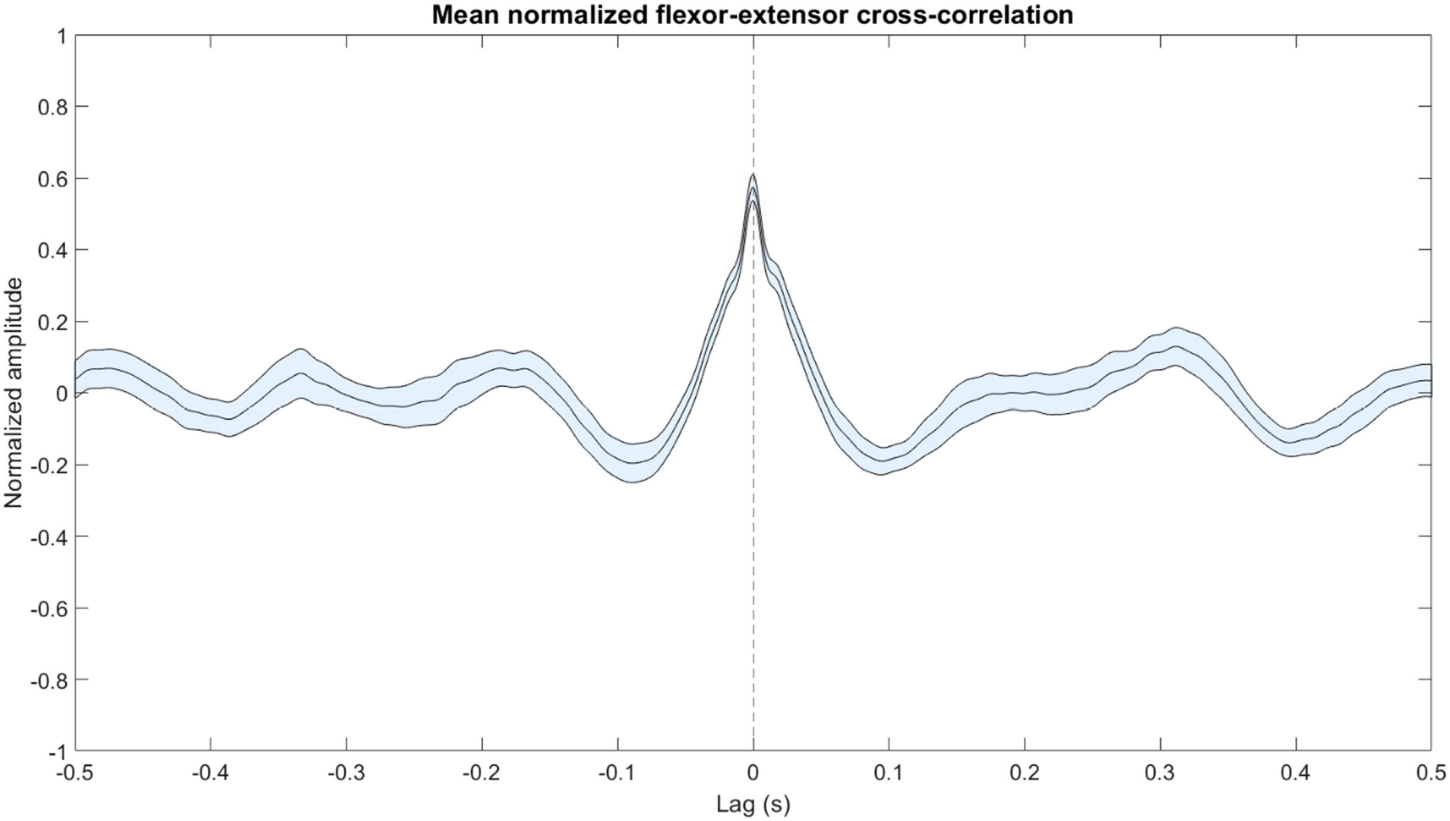
Average normalized cross‐correlation of tremor activity in wrist flexor and extensor EMG signals. The blue shaded region shows the 95% confidence interval. *N* = 66 pairs.

Tremor was observed clinically and in the EMG data on both sides. Figure 4 shows a typical example of a TC recorded for 80s at 4.29 mm above target. The left panel (4a) shows that in the time domain, the extracellular spikes correspond with bursts in both EMG channels. We observe a clear periodicity of 200–250 ms (Figure 4b) and a peak in power at 4–5 Hz (Figure 4c) consistent with tremor activity. Figure 4d shows that all SPK–EMG and EMG–EMG pairs are synchronized (i.e., the phase delay is approximately zero), although in the other two tremor cells that were observed the spiking activity was in an alternating pattern with the EMG tremor activity (i.e., an approximately 180 degree lag). Both phase lags observed are consistent with previous reports (Zakaria et al., 2013) which show a wide range of potential lags between spiking and EMG tremor activity in essential tremor. Figure 4e shows a high neuronal/EMG coherence at the tremor frequency in the theta band (4–5 Hz). Figure 5 shows that recordings from wrist flexor and extensor EMG consistently showed highly synchronous tremor activity.

In the operating room, we stimulated the macro‐electrode at 3 mm above the preplanned target on the left side with an amplitude of 0.25 mA, resulting in full tremor arrest in the right hand. We did not observe strong tremor in the left hand before testing the clinical effects of stimulation (possibly due to a micro‐lesion effect), and therefore we could not verify tremor arrest. In both hemispheres the patient experienced slight transient paresthesia in his contralateral hand at 1 mA stimulation. No other side effects were observed up to 4 mA.

On postoperative follow‐up, the IPG was programmed without stimulation‐induced side effects, with active contacts three (second from top) on the left and four (top) on the right (Figure 3). The patient experienced robust improvement in tremor with a 3 month postoperative TETRAS score of 16 (76% improvement).

## 5 DISCUSSION

Our results show that indirect targeting of the Vim in patients with MRI‐incompatible devices can be successfully achieved using contrast CT imaging assisted by electrophysiological recordings. While inter‐patient anatomical variability presents a challenge to successful indirect targeting of the Vim, the use of multiunit MERs with high spatial sampling can greatly assist in successfully locating the Vim. The steep rise in RMS (background activity) and the presence of high‐amplitude spikes (Ohye & Narabayashi, 1979), spectral activity in the tremor frequency (4–7 Hz), and TCs, show that the identification of the Vim can be achieved successfully using short duration (8 s), densely sampled (100 μm interval) multiunit recordings.

While the Lead‐DBS results suggest that the lead entered the Vim on the left side 1–2 mm higher than the start of the steep rise in NRMS observed in the electrophysiology (Figure 3), this is may be due to the limited resolution of the CT scan (1 mm). The relative proximity of the right lead to the Vc (as compared to the left lead) may explain why we did not detect TCs in the right trajectory despite passing through the Vim (Gross et al., 2006). Ultimately, the decision that DBS leads are optimally located is obtained by careful balance between electrophysiology, assessment of the therapeutic window of stimulation, and postoperative imaging. The TC results show high power in the tremor frequency at 4–5 Hz (in the lower range of tremor frequencies in ET; Deuschl et al., 1998) in both extracellular and EMG recordings. The synchronous behavior of the flexor and extensor EMG channels is usually (although not always) seen in ET as opposed to the alternating pattern (phase lag of 180 degrees) more typical of Parkinson's (Hallett, 1998), and is therefore consistent with the patient's final diagnosis (Milanov, 2000). However, this distinction is disputed (Thenganatt & Louis, 2012).

In summary, we successfully managed to locate the AC and PC, and the Vim using CT with contrast media combined with electrophysiology and assessment of clinical outcomes in a patient with an MRI‐incompatible pacemaker lead.

## AUTHOR CONTRIBUTIONS

Stefanie Glowinsky and Hagai Bergman assisted in real time physiology, analyzed data, prepared figures, and drafted manuscript; Omer Zarchi performed real‐time physiology; Shlomo Fireman was anesthesiologist; Johnathan Reiner performed neurological assessment before and after surgery; Idit Tamir performed surgery, clinical evaluation, edited and revised manuscript; Stefanie Glowinsky, Hagai Bergman, and Idit Tamir wrote the manuscript; all authors read and approved final version of manuscript.

## FUNDING INFORMATION

Stefanie Glowinsky is a fellow of the Ariane de Rothschild Women's Doctoral Program. This study was partially supported by the using DBS electrophysiology to optimize clinical outcome of GBA Parkinson's patients grant of the Silverstein foundation (to HB).

## CONFLICT OF INTEREST STATEMENT

No conflicts of interest, financial or otherwise, are declared by the authors.

## ETHICS STATEMENT

We obtained written informed consent from the patient for the study.

## Data Availability

Original data and Matlab code will be available by request from the corresponding author.
